# Supplementary material for: Comparison of Illumina and Oxford Nanopore Technology systems for the genomic characterization of Streptococcus pneumoniae
Source: Microbiol Spectr. 2025 May 28;13(7):e01294-24. doi: 10.1128/spectrum.01294-24 (PMC12210953; doi:10.1128/spectrum.01294-24)
Supplement: Supplemental tables and figures — Tables S1 to S8, and Fig. S1 and S2. [file spectrum.01294-24-s0001.docx]

**Comparison of Illumina and Oxford Nanopore Technology systems for the genomic characterization of *Streptococcus pneumoniae***

**Supplemental Material**

**I. Tables**

**Table S1: The demographic and clinical characteristics of patients with invasive pneumococcal disease with a sample sequenced using Illumina and ONT_V10 technologies.**

|  | **Patients with invasive  pneumococcal disease (n=27)** |
| --- | --- |
| **Males*** | 13/26 (50 %) |
| **Age** |  |
| **Age ≤5years** | 13 (48 %) |
| **5> Age <65 years** | 8 (30 %) |
| **Age≥ 65 years** | 6 (22 %) |
| **Region** |  |
| **Beirut** | 15 (56 %) |
| **Mount Lebanon** | 8 (30 %) |
| **South Lebanon** | 2 (7 %) |
| **North Lebanon** | 2 (7 %) |
| **Year** |  |
| **2017** | 1 (4 %) |
| **2018** | 3 (11 %) |
| **2022** | 11 (41 %) |
| **2023** | 12 (44 %) |
| **Clinical Manifestation*** |  |
| **Pneumonia** | 11/25 (44 %) |
| **Bacteremia** | 7/25 (28 %) |
| **Meningitis** | 4/25 (16 %) |
| **Other** | 3/25 (12 %) |

*Data was missing for some specimens

**Table S2: Summary statistics of raw data (n=27) generated by Illumina and ONT_V10.**

| **Sample** | **Bases** | **Sequencing Depth (X)** | **Total Reads** | **Read Length (bp);  mean (SD)** | **Max Read Length (bp); mean (SD)** | **Quality Scores; mean (SD)** |
| --- | --- | --- | --- | --- | --- | --- |
| **S515_ONT_V10** | 239,468,234 | 108.84 | 249,443 | 960 (1322) | 22,138 | 18.25 (2.45) |
| **S515_Illumina** | 115,138,526 | 52.54 | 485,190 | 238 (36) | 251 | 33.89 (4.42) |
| **S516_ONT_V10** | 87,818,345 | 39.91 | 158,290 | 554 (679) | 53,307 | 17.56 (2.53) |
| **S516_Illumina** | 161,768,287 | 124.36 | 1,287,316 | 212 (48) | 251 | 35.15 (3.79) |
| **S524_ONT_V10** | 231,292,288 | 105.13 | 161,339 | 1433 (1665) | 19,033 | 18.49 (2.37) |
| **S524_Illumina** | 202,774,087 | 92.41 | 868,878 | 234 (37.5) | 251 | 35.38 (3.1) |
| **S531_ONT_V10** | 82,233,245 | 37.37 | 130,153 | 631 (627) | 17,469 | 19.08 (2.71) |
| **S531_Illumina** | 272,288,956 | 73.75 | 681,266 | 238 (34) | 251 | 34.64 (3.58) |
| **S644_ONT_V10** | 464,664,375 | 211.21 | 640,641 | 725 (992) | 61,277 | 19.46 (2.77) |
| **S644_Illumina** | 224,237,947 | 102.38 | 1,110,034 | 202 (50) | 251 | 35.54 (3.06) |
| **S645_ONT_V10** | 105,187,738 | 47.81 | 181,157 | 580 (844) | 44,027 | 18.2 (2.62) |
| **S645_Illumina** | 186,630,015 | 85.38 | 812,388 | 231 (40) | 251 | 34.76 (3.77) |
| **S647_ONT_V10** | 140,343,327 | 63.79 | 194,281 | 722 (892) | 51,177 | 18.26 (2.64) |
| **S647_Illumina** | 188,740,320 | 86.06 | 814,302 | 232 (39) | 251 | 35.12 (3.45) |
| **S649_ONT_V10** | 669,464,805 | 304.3 | 662,443 | 1010 (1434) | 57,097 | 19.54 (2.73) |
| **S649_Illumina** | 212,980,898 | 97.21 | 945,154 | 226 (43) | 251 | 35.09 (3.55) |
| **S650_ONT_V10** | 797,699,969 | 362.59 | 789,298 | 1010 (1151) | 58,457 | 19.76 (2.75) |
| **S650_Illumina** | 130,405,334 | 59.47 | 542,966 | 240 (32) | 251 | 34.85 (3.55) |
| **S651_ONT_V10** | 407,958,961 | 185.43 | 570,727 | 714 (935) | 49,256 | 19.46 (2.75) |
| **S651_Illumina** | 168,902,855 | 77.08 | 710,518 | 238 (35) | 251 | 35.11 (3.62) |
| **S653_ONT_V10** | 90,694,044 | 41.22 | 142,476 | 636 (1117) | 54,142 | 18.36 (2.6) |
| **S653_Illumina** | 224,401,541 | 102.38 | 1,000,600 | 225 (43) | 251 | 35 (3.52) |
| **S660_ONT_V10** | 373,800,121 | 169.9 | 267,675 | 1396 (2961) | 86,561 | 19.31 (2.7) |
| **S660_Illumina** | 147,504,201 | 67.36 | 645,194 | 229 (39) | 251 | 34.3 (3.4) |
| **S664_ONT_V10** | 125,558,567 | 57.07 | 198,031 | 634 (1182) | 288,361 | 18.14 (2.66) |
| **S664_Illumina** | 149,160,372 | 68 | 659,424 | 226 (41) | 251 | 35.55 (3.02) |
| **S666_ONT_V10** | 140,237,176 | 63.74 | 171,687 | 816 (1399) | 61,559 | 19.07 (2.71) |
| **S666_Illumina** | 189,452,824 | 86.36 | 823,426 | 230 (41) | 251 | 35.42 (3.44) |
| **S668_ONT_V10** | 106,293,393 | 48.31 | 175,487 | 605 (995) | 56,105 | 17.86 (2.62) |
| **S668_Illumina** | 168,966,696 | 77.29 | 726,678 | 234 (39) | 251 | 35.16 (3.5) |
| **S669_ONT_V10** | 90,276,872 | 41.03 | 112,064 | 805 (1391) | 62,987 | 19.07 (2.72) |
| **S669_Illumina** | 114,449,029 | 52.16 | 484,630 | 236.8 (38) | 251 | 35.07 (3.6) |
| **S670_ONT_V10** | 129,463,991 | 58.84 | 157,854 | 820 (1321) | 52,875 | 19.06 (2.71) |
| **S670_Illumina** | 150,912,735 | 69.14 | 639,484 | 237 (35) | 251 | 34.07 (4.19) |
| **S673_ONT_V10** | 126,833,661 | 57.65 | 188,940 | 671 (1044) | 52,239 | 18.22 (2.69) |
| **S673_Illumina** | 93,752,317 | 43.13 | 392,404 | 241 (31) | 251 | 32.99 (4.8) |
| **S674_ONT_V10** | 159,014,948 | 72.27 | 229,114 | 694 (1263) | 60,511 | 18.21 (2.63) |
| **S674_Illumina** | 229,193,582 | 104.77 | 979,612 | 235 (36) | 251 | 35.22 (3.5) |
| **S676_ONT_V10** | 169,241,760 | 76.92 | 248,750 | 680 (1167) | 63,128 | 18.25 (2.65) |
| **S676_Illumina** | 127,796,121 | 58.45 | 542,298 | 237 (38) | 251 | 34.77 (3.71) |
| **S678_ONT_V10** | 92,790,854 | 42.17 | 157,118 | 590 (813) | 42,436 | 18.95 (2.7) |
| **S678_Illumina** | 143,466,386 | 65.95 | 609,096 | 238 (34) | 251 | 32.87 (4.98) |
| **S682_ONT_V10** | 113,526,277 | 51.6 | 156,222 | 726 (1126) | 86,796 | 18.9 (2.72) |
| **S682_Illumina** | 189,780,763 | 86.77 | 803,326 | 237 (35) | 251 | 34.88 (3.68) |
| **S683_ONT_V10** | 101,022,665 | 45.91 | 115,896 | 871 (1987) | 70,153 | 19.19 (2.73) |
| **S683_Illumina** | 171,946,225 | 78.84 | 749,322 | 231 (39) | 251 | 34.76 (3.35) |
| **S685_ONT_V10** | 156,473,906 | 71.12 | 234,770 | 666 (1257) | 54,115 | 17.99 (2.64) |
| **S685_Illumina** | 268,274,397 | 122.33 | 1,147,722 | 234 (39) | 251 | 35.04 (3.56) |
| **S686_ONT_V10** | 93,112,035 | 42.32 | 112,992 | 824 (1283) | 64,389 | 19.1 (2.67) |
| **S686_Illumina** | 144,457,877 | 65.85 | 608,174 | 238 (34) | 251 | 35.07 (3.54) |
| **S688_ONT_V10** | 119,171,455 | 54.16 | 131,275 | 907 (1553) | 62,665 | 19.22 (2.73) |
| **S688_Illumina** | 112,618,354 | 51.44 | 471,922 | 239 (32) | 251 | 34.5 (3.94) |
| **S689_ONT_V10** | 557,700,759 | 253.5 | 789,068 | 706 (798) | 57,062 | 19.51 (2.73) |
| **S689_Illumina** | 184,309,185 | 84.05 | 779,812 | 237 (36) | 251 | 35.15 (3.55) |

Abbreviations: bp, base pairs; ONT_V10, Oxford Nanopore Technologies (R9.4.1 flowcells/ Kit10 chemistry); SD, Standard deviation

**Table S3: A comparison of assembly QC and GPSC data between the assembleBAC-ONT and canu outputs for long-read sequencing analysis.**

|  | **Number of Contigs** | | **Genome Size** | | **GPSC strain** | |
| --- | --- | --- | --- | --- | --- | --- |
| **Isolate** | **porechop/canu** | **assembleBAC-ONT** | **porechop/canu** | **assembleBAC-ONT** | **porechop/canu** | **assembleBAC-ONT** |
| **S515** | 46 | 1 | **2,434,833** | 2,070,569 | 19 | 19 |
| **S516** | 76 | 80 | 2,102,196 | 1,954,808 | Not assigned | 12 |
| **S524** | 48 | 1 | **2,491,187** | 2,113,477 | Not assigned | 61 |
| **S531** | 231 | 276 | 2,199,404 | 1,974,716 | Not assigned | 31 |
| **S644** | 23 | 1 | 2,150,789 | 2,002,161 | 3 | 3 |
| **S645** | 276 | 241 | 2,179,097 | 2,023,275 | Not assigned | 7 |
| **S647** | 66 | 52 | 2,269,160 | 2,097,852 | Not assigned | 67 |
| **S649** | 22 | 2 | **2,604,526** | 2,376,303 | 38 | 38 |
| **S650** | 40 | 1 | 2,352,969 | 1,997,765 | 3 | 3 |
| **S651** | 21 | 1 | 2,109,066 | 1,996,333 | 12 | 12 |
| **S653** | 152 | 187 | 2,191,055 | 2,075,588 | Not assigned | 10 |
| **S660** | 17 | 2 | **2,733,669** | 2,186,015 | Not assigned | 6 |
| **S664** | 65 | 79 | 2,304,947 | 2,179,516 | 10 | 10 |
| **S666** | 29 | 10 | 2,125,338 | 2,025,396 | 3 | 3 |
| **S668** | N/A* | 152 | N/A | 2,076,054 |  | 8 |
| **S669** | 55 | 91 | 2,181,472 | 2,133,451 | Not assigned | Not assigned |
| **S670** | 18 | 2 | 2,207,539 | 2,106,665 | Not assigned | 5 |
| **S673** | 52 | 68 | 2,130,924 | 2,039,203 | 3 | 3 |
| **S674** | 39 | 14 | 2,260,880 | 2,128,320 | 10 | 10 |
| **S676** | 48 | 20 | 2,325,265 | 2,187,386 | 10 | 10 |
| **S678** | 87 | 172 | 2,215,842 | 2,023,064 | 699 | 699 |
| **S682** | 34 | 15 | 2,212,309 | 2,146,442 | 10 | 10 |
| **S683** | 18 | 26 | 2,172,541 | **2,436,413** | Not assigned | Not assigned |
| **S685** | 67 | 81 | 2,364,294 | **2,964,848** | Not assigned | Not assigned |
| **S686** | 33 | 54 | 2,207,822 | 2,124,037 | Not assigned | 10 |
| **S688** | 39 | 1 | 2,280,200 | 2,092,159 | Not assigned | 35 |
| **S689** | 44 | 5 | **2,575,883** | 2,326,859 | 38 | 38 |

All isolates were sequenced using the Oxford Nanopore Technologies with R9.4.1 flowcells and Kit10 chemistry (ONT_V10). A genome size between 1.9 - 2.4 Mb was required to pass Assembly QC.

Abbreviations: GPSC, Global pneumococcal sequence clusters; QC, Quality control

**Table S4: Quality measures of assemblies based on data from the Illumina MiSeq and ONT MinION Mk1C systems.**

| **Sample** | **Genome Size (bp)** | **GC-content (%)** | **Contigs #** | **N50** |
| --- | --- | --- | --- | --- |
| **S515_ONT_V10** | 2,070,569 | 39.6 | 1 | 2,070,569 |
| **S515_Illumina** | 2,031,162 | 39.6 | 41 | 83,922 |
| **S516_ONT_V10** | 1,954,808 | 39.8 | 80 | 44,982 |
| **S516_Illumina** | 1,970,432 | 39.8 | 21 | 289,540 |
| **S524_ONT_V10** | 2,113,477 | 39.6 | 1 | 2,113,477 |
| **S524_Illumina** | 2,086,537 | 39.5 | 33 | 143,846 |
| **S531_ONT_V10** | 1,974,716 | 39.5 | 276 | 9,928 |
| **S531_Illumina** | 2,078,512 | 39.5 | 57 | 60,275 |
| **S644_ONT_V10** | 2,002,161 | 39.8 | 1 | 2,002,161 |
| **S644_Illumina** | 1,978,651 | 39.7 | 28 | 121,097 |
| **S645_ONT_V10** | 2,023,275 | 39.4 | 241 | 12,506 |
| **S645_Illumina** | 2,050,654 | 39.6 | 40 | 95,402 |
| **S647_ONT_V10** | 2,097,852 | 39.5 | 52 | 76,148 |
| **S647_Illumina** | 2,074,775 | 39.7 | 43 | 166,683 |
| **S649_ONT_V10** | 2,376,303 | 39.4 | 2 | 2,369,396 |
| **S649_Illumina** | 2,286,318 | 39.4 | 93 | 45,132 |
| **S650_ONT_V10** | 1,997,765 | 39.8 | 1 | 1,997,765 |
| **S650_Illumina** | 1,969,753 | 39.8 | 29 | 175,894 |
| **S651_ONT_V10** | 1,996,333 | 39.9 | 1 | 1,996,333 |
| **S651_Illumina** | 1,970,435 | 39.8 | 21 | 289,542 |
| **S653_ONT_V10** | 2,075,588 | 39.6 | 187 | 16,912 |
| **S653_Illumina** | 2,093,729 | 39.7 | 47 | 148,749 |
| **S660_ONT_V10** | 2,186,015 | 39.7 | 2 | 2,183,834 |
| **S660_Illumina** | 2,126,629 | 39.6 | 58 | 83,146 |
| **S664_ONT_V10** | 2,179,516 | 39.4 | 79 | 52,290 |
| **S664_Illumina** | 2,183,134 | 39.5 | 50 | 128,140 |
| **S666_ONT_V10** | 2,025,389 | 39.7 | 10 | 1,167,421 |
| **S666_Illumina** | 1,989,880 | 39.7 | 29 | 111,103 |
| **S668_ONT_V10** | 2,076,054 | 39.6 | 152 | 21,554 |
| **S668_Illumina** | 2,099,911 | 39.6 | 40 | 129,271 |
| **S669_ONT_V10** | 2,133,451 | **40.2** | 91 | 44,202 |
| **S669_Illumina** | 2,061,162 | 39.8 | 30 | 131,691 |
| **S670_ONT_V10** | 2,106,665 | 39.8 | 2 | 2,105,287 |
| **S670_Illumina** | 2,068,994 | 39.7 | 32 | 127,918 |
| **S673_ONT_V10** | 2,039,203 | 39.6 | 68 | 59,228 |
| **S673_Illumina** | 2,021,607 | 39.7 | 30 | 128,382 |
| **S674_ONT_V10** | 2,128,320 | 39.5 | 14 | 236,567 |
| **S674_Illumina** | 2,111,019 | 39.5 | 46 | 134,073 |
| **S676_ONT_V10** | 2,187,386 | 39.5 | 20 | 196,047 |
| **S676_Illumina** | 2,154,603 | 39.5 | 35 | 214,778 |
| **S678_ONT_V10** | 2,030,894 | 39.6 | 170 | 18,820 |
| **S678_Illumina** | 2,080,915 | 39.6 | 67 | 64,505 |
| **S682_ONT_V10** | 2,146,442 | 39.6 | 15 | 299,441 |
| **S682_Illumina** | 2,112,393 | 39.5 | 44 | 124,894 |
| **S683_ONT_V10** | **2,436,413** | **42.2** | 26 | 347,144 |
| **S683_Illumina** | 2,104,717 | 39.6 | 49 | 84,459 |
| **S685_ONT_V10** | **2,964,848** | **45.2** | 81 | 61,365 |
| **S685_Illumina** | 2,167,458 | 39.6 | 50 | 124,632 |
| **S686_ONT_V10** | 2,124,037 | 39.5 | 54 | 99,075 |
| **S686_Illumina** | 2,105,938 | 39.5 | 34 | 151,641 |
| **S688_ONT_V10** | 2,092,159 | 39.6 | 1 | 2,092,159 |
| **S688_Illumina** | 2,058,613 | 39.6 | 40 | 107,917 |
| **S689_ONT_V10** | 2,326,859 | 39.4 | 5 | 1,521,710 |
| **S689_Illumina** | 2,229,195 | 39.4 | 99 | 39,823 |

Abbreviations: bp, base pairs; GC-content, guanine-cytosine content; ONT_V10, Oxford Nanopore Technologies (R9.4.1 flowcells/ Kit10 chemistry)

**Table S5:** Penicillin-binding protein types, penicillin resistance and MIC predicted by Illumina and ONT_V10 in *S. pneumoniae* isolates (n=27) causing invasive disease.

|  | **Penicillin-binding protein types** | | |  |  |  |
| --- | --- | --- | --- | --- | --- | --- |
| **Isolate** | **pbp1a** | **pbp2b** | **pbp2x** | **Penicillin** | **Predicted MIC** | **AST** |
| **S515_ONT_V10** | 1 | 2 | New | S | ≤0.03 | S |
| **S515_Illumina** | 1 | 2 | 2 | S | ≤0.03 |  |
| **S516_ONT_V10** | 2 | 0 | 2 | S | ≤0.03 | S |
| **S516_Illumina** | 2 | 0 | 2 | S | ≤0.03 |  |
| **S524_ONT_V10** | 0 | 53 | New | R | 0.12 | R |
| **S524_Illumina** | 0 | 53 | 77 | R | 0.12 |  |
| **S531_ONT_V10** | 23 | Nf | 5 | - | - | S |
| **S531_Illumina** | 23 | 6 | 5 | S | ≤0.03 |  |
| **S644_ONT_V10** | 3 | 6 | 5 | S | ≤0.03 | S |
| **S644_Illumina** | 3 | 6 | 5 | S | ≤0.03 |  |
| **S645_ONT_V10** | Nf | Nf | New | - | - | N/A |
| **S645_Illumina** | 2 | 0 | New | R | 0.12 |  |
| **S647_ONT_V10** | 84 | New | New | R | 0.25 | R |
| **S647_Illumina** | 84 | 141 | 229 | R | 0.25 |  |
| **S649_ONT_V10** | New | 1 | 77 | R | 0.12 | R |
| **S649_Illumina** | New | 1 | 77 | R | 0.12 |  |
| **S650_ONT_V10** | 3 | 0 | New | S | ≤0.03 | S |
| **S650_Illumina** | 3 | 0 | 2 | S | ≤0.03 |  |
| **S651_ONT_V10** | 2 | 0 | New | S | ≤0.03 | S |
| **S651_Illumina** | 2 | 0 | 2 | S | ≤0.03 |  |
| **S653_ONT_V10** | 79 | error | 171 | R | **0.25** | R |
| **S653_Illumina** | 79 | 43 | 171 | R | **0.12** |  |
| **S660_ONT_V10** | New | 12 | 18 | R | 2 | R |
| **S660_Illumina** | 31 | 12 | 18 | R | 4 |  |
| **S664_ONT_V10** | New | Nf | New | - | - | R |
| **S664_Illumina** | 17 | 15 | 367 | R | 0.25 |  |
| **S666_ONT_V10** | 150 | 53 | 77 | R | 0.12 | N/A |
| **S666_Illumina** | 150 | 53 | 77 | R | 0.12 |  |
| **S668_ONT_V10** | 23 | 0 | error | S | 0.06 | S |
| **S668_Illumina** | 23 | 0 | 21 | S | ≤0.03 |  |
| **S669_ONT_V10** | 2 | 4 | 77 | S | 0.06 | S |
| **S669_Illumina** | 2 | 4 | 77 | S | 0.06 |  |
| **S670_ONT_V10** | New | 1 | error | R | **0.25** | R |
| **S670_Illumina** | New | 1 | New | R | **0.5** |  |
| **S673_ONT_V10** | 2 | 0 | 6 | S | ≤0.03 | S |
| **S673_Illumina** | 2 | 0 | 6 | S | ≤0.03 |  |
| **S674_ONT_V10** | 17 | 15 | New | R | 0.25 | R |
| **S674_Illumina** | 17 | 15 | 367 | R | 0.25 |  |
| **S676_ONT_V10** | 17 | 15 | error | R | 0.25 | R |
| **S676_Illumina** | 17 | 15 | 367 | R | 0.25 |  |
| **S678_ONT_V10** | error | error | 8 | - | - | R |
| **S678_Illumina** | 17 | 144 | 8 | R | 2 |  |
| **S682_ONT_V10** | Nf | error | 205 | - | - | R |
| **S682_Illumina** | 17 | 16 | 205 | R | 2 |  |
| **S683_ONT_V10** | 10 | error | New | R | 0.5 | R |
| **S683_Illumina** | 10 | 9 | New | R | 0.5 |  |
| **S685_ONT_V10** | 34 | 32 | 43 | R | 2 | R |
| **S685_Illumina** | 34 | 32 | 43 | R | 2 |  |
| **S686_ONT_V10** | error | 15 | 22 | R | 0.5 | R |
| **S686_Illumina** | 17 | 15 | 22 | R | 0.5 |  |
| **S688_ONT_V10** | error | 0 | 53 | - | - | S |
| **S688_Illumina** | 0 | 0 | 53 | S | ≤0.03 |  |
| **S689_ONT_V10** | 150 | 1 | New | R | 0.12 | R |
| **S689_Illumina** | 150 | 1 | 77 | R | 0.12 |  |

Predicted penicillin MIC was interpreted using CLSI guidelines (2014, M100-S24) based on the meningitis MIC cut-off. Penicillin with MIC>0.06 µg/ml was categorized as resistant.

Abbreviations: pbp, Penicillin-binding protein; MIC, Minimum inhibitory concentration; Nf, Not found; ONT_V10, Oxford Nanopore Technologies (R9.4.1 flow cells/ Kit10 chemistry); R, Resistant; S, Sensitive

**Table S6:** Penicillin-binding protein types, penicillin resistance and MIC predicted by ONT_V14 in *S. pneumoniae* isolates (n=9) causing invasive disease.

|  | **Penicillin-binding protein types** | | |  |  |
| --- | --- | --- | --- | --- | --- |
| **Isolate** | **pbp1a** | **pbp2b** | **pbp2x** | **Penicillin** | **Predicted MIC** |
| **S645_ONT_V10** | Nf | Nf | New | - | - |
| **S645_ONT_V14** | 2 | 0 | New | R | 0.12 |
| **S647_ONT_V10** | 84 | New | New | R | 0.25 |
| **S647_ONT_V14** | 84 | New | 229 | R | 0.25 |
| **S650_ONT_V10** | 3 | 0 | New | S | ≤0.03 |
| **S650_ONT_V14** | 3 | 0 | 2 | S | ≤0.03 |
| **S653_ONT_V10** | 79 | error | 171 | R | 0.25 |
| **S653_ONT_V14** | 79 | 43 | 171 | R | 0.12 |
| **S664_ONT_V10** | New | Nf | New | - | - |
| **S664_ONT_V14** | 17 | 15 | Nf | - | - |
| **S668_ONT_V10** | 23 | 0 | error | S | 0.06 |
| **S668_ONT_V14** | 23 | 0 | 21 | S | ≤0.03 |
| **S670_ONT_V10** | New | 1 | error | R | 0.25 |
| **S670_ONT_V14** | New | 1 | New | R | 0.5 |
| **S678_ONT_V10** | error | error | 8 | - | - |
| **S678_ONT_V14** | 17 | 144 | 8 | R | 2 |
| **S683_ONT_V10** | 10 | error | New | R | 0.5 |
| **S683_ONT_V14** | 10 | 9 | New | R | 0.5 |

Predicted penicillin MIC was interpreted using CLSI guidelines (2014, M100-S24) based on the meningitis MIC cut-off. Penicillin with MIC>0.06 µg/ml was categorized as resistant.

Abbreviations: pbp, Penicillin-binding protein; MIC, Minimum inhibitory concentration; Nf, Not found; ONT_V10, Oxford Nanopore Technologies (R9.4.1 flow cells/ Kit10 chemistry); ONT_V14, Oxford Nanopore Technologies (R10.4.1 flow cells/ Kit14 chemistry); R, Resistant; S, Sensitive

**Table S7:** Quality of genomes generated by short-read-first hybrid assembly using Unicycler or long-read-first assembly followed by short-read polishing using Pilon. Genomes with initial ONT_V10 sequencing depth >100X (n=8) are shown below.

| **Genome** | **ONT_V10 Depth** | **Genome Length (bp)** | **Contigs #** | **Largest Contig (bp)** | **N50** | **GC-Content (%)** |
| --- | --- | --- | --- | --- | --- | --- |
| **Uni_515** | 108.8X | 2,069,323 | 1 | 2,069,323 | 2,069,323 | 39.7 |
| **Pilon_515** |  | 2,069,963 | 1 | 2,069,963 | 2,069,963 | 39.7 |
| **Uni_524** | 105X | 2,112,002 | 1 | 2,112,002 | 2,112,002 | 39.6 |
| **Pilon_524** |  | 2,112,871 | 1 | 2,112,871 | 2,112,871 | 39.6 |
| **Uni_644** | 211X | 2,000,989 | 1 | 2,000,989 | 2,000,989 | 39.8 |
| **Pilon_644** |  | 2,001,146 | 1 | 2,001,146 | 2,001,146 | 39.8 |
| **Uni_649** | 304X | 2,371,217 | **6** | 2,345,109 | 2,345,109 | 39.5 |
| **Pilon_649** |  | 2,375,130 | **2** | 2,368,230 | 2,368,230 | 39.5 |
| **Uni_650** | 362X | 1,990,462 | **30** | 850,787 | **659,445** | 39.8 |
| **Pilon_650** |  | 1,996,715 | **1** | 1,996,715 | **1,996,715** | 39.8 |
| **Uni_651** | 185X | 1,995,166 | 1 | 1,995,166 | 1,995,166 | 39.9 |
| **Pilon_651** |  | 1,995,357 | 1 | 1,995,357 | 1,995,357 | 39.9 |
| **Uni_660** | 169X | 2,182,741 | **6** | 2,144,503 | 2,144,503 | 39.7 |
| **Pilon_660** |  | 2,185,195 | **2** | 2,183,014 | 2,183,014 | 39.7 |
| **Uni_689** | 253X | 2,291,473 | **45** | 948,671 | **448,211** | 39.4 |
| **Pilon_689** |  | 2,325,675 | **5** | 1,520,944 | **1,520,944** | 39.4 |

Only genomes sequenced with ONT_V10 (R9.4.1 flowcells/ Kit10 chemistry) were utilized for hybrid assembly.

Abbreviations: bp, base pairs; GC-content, guanine-cytosine content; Uni, Unicycler

**Table S8:** Quality of genomes generated by short-read-first hybrid assembly using Unicycler or long-read-first assembly followed by short-read polishing using Pilon. The below genomes have an initial ONT sequencing depth <100X (n=19).

| **Genome** | **ONT_V10 Depth** | **Genome Length (bp)** | **Contigs #** | **Largest Contig (bp)** | **N50** | **GC-Content (%)** | **Missing MLST alleles** |
| --- | --- | --- | --- | --- | --- | --- | --- |
| **Uni_516** | 39.9X | 1,977,674 | 15 | 1,626,840 | 1,626,840 | 39.8 |  |
| **Pilon_516** |  | 1,953,868 | 80 | 131,724 | 44,967 | 39.8 |  |
| **Uni_531** | 37X | 2,104,174 | 47 | 419,480 | 222,439 | 39.5 |  |
| **Pilon_531** |  | 1,974,046 | 276 | 63,822 | 9,923 | 39.5 |  |
| **Uni_645** | 47.8X | 2,056,914 | 38 | 488,257 | 274,038 | 39.6 |  |
| **Pilon_645** |  | 2,021,343 | 241 | 44,898 | 12,495 | 39.6 | 2 |
| **Uni_647** | 63.7X | 2,083,730 | 36 | 1,559,312 | 1,559,312 | 39.7 |  |
| **Pilon_647** |  | 2,095,898 | 52 | 294,217 | 76,081 | 39.7 | 1 |
| **Uni_653** | 41X | 2,102,956 | 51 | 652,056 | 223,080 | 39.6 |  |
| **Pilon_653** |  | 2,074,188 | 187 | 49,353 | 16,906 | 39.6 | 1 |
| **Uni_664** | 57X | 2,203,748 | 38 | 1,295,319 | 1,295,319 | 39.5 |  |
| **Pilon_664** |  | 2,178,282 | 79 | 167,994 | 52,260 | 39.5 | 1 |
| **Uni_666** | 63X | 2,004,310 | 22 | 1,179,839 | 1,179,839 | 39.7 |  |
| **Pilon_666** |  | 2,024,351 | 10 | 1,166,834 | 1,166,834 | 39.7 |  |
| **Uni_668** | 48X | 2,123,128 | 20 | 2,051,267 | 2,051,267 | 39.7 |  |
| **Pilon_668** |  | 2,074,823 | 152 | 104,773 | 21,546 | 39.6 |  |
| **Uni_669** | 41X | 2,062,503 | 40 | 804,790 | 362,629 | 39.8 |  |
| **Pilon_669** |  | 2,132,231 | 91 | 94,632 | 44,178 | 40.2 |  |
| **Uni_670** | 58X | 2,095,499 | 35 | 918,842 | 708,881 | 39.8 |  |
| **Pilon_670** |  | 2,105,624 | 2 | 2,104,246 | 2,104,246 | 39.9 |  |
| **Uni_673** | 57.6X | 2,027,492 | 13 | 1,686,246 | 1,686,246 | 39.7 |  |
| **Pilon_673** |  | 2,038,206 | 68 | 200,210 | 59,186 | 39.6 |  |
| **Uni_674** | 72X | 2,122,551 | 21 | 1,322,604 | 1,322,604 | 39.5 |  |
| **Pilon_674** |  | 2,127,241 | 14 | 389,477 | 236,474 | 39.5 |  |
| **Uni_676** | 76X | 2,161,287 | 29 | 1,214,963 | 1,214,963 | 39.5 |  |
| **Pilon_676** |  | 2,186,264 | 20 | 714,370 | 195,943 | 39.5 |  |
| **Uni_678** | 42X | 2,113,886 | 46 | 280,856 | 222,928 | 39.7 |  |
| **Pilon_678** |  | 2,029,702 | 170 | 63,861 | 18,815 | 39.6 | 1 |
| **Uni_682** | 51X | 2,118,100 | 31 | 1,205,502 | 1,205,502 | 39.4 |  |
| **Pilon_682** |  | 2,145,176 | 15 | 795,799 | 299,279 | 39.6 |  |
| **Uni_683** | 45.9X | 2,125,020 | 2 | 2,104,023 | 2,104,023 | 39.6 |  |
| **Pilon_683** |  | 2,435,185 | 26 | 1,006,796 | 346,903 | 42.2 |  |
| **Uni_685** | 71X | 2,182,170 | 48 | 960,746 | 662,898 | 39.6 |  |
| **Pilon_685** |  | 2,963,674 | 81 | 202,689 | 61,365 | 45.2 |  |
| **Uni_686** | 42X | 2,114,274 | 36 | 1,210,306 | 1,210,306 | 39.4 |  |
| **Pilon_686** |  | 2,122,785 | 54 | 366,269 | 99,014 | 39.5 |  |
| **Uni_688** | 54X | 2,090,320 | 15 | 1,999,282 | 1,999,282 | 39.6 |  |
| **Pilon_688** |  | 2,091,090 | 1 | 2,091,090 | 2,091,090 | 39.6 |  |

Only genomes sequenced with ONT_V10 (R9.4.1 flowcells/ Kit10 chemistry) were utilized for hybrid assembly.

Abbreviations: bp, base pairs; GC-content, guanine-cytosine content; Uni, Unicycler

**II. Figures**

**
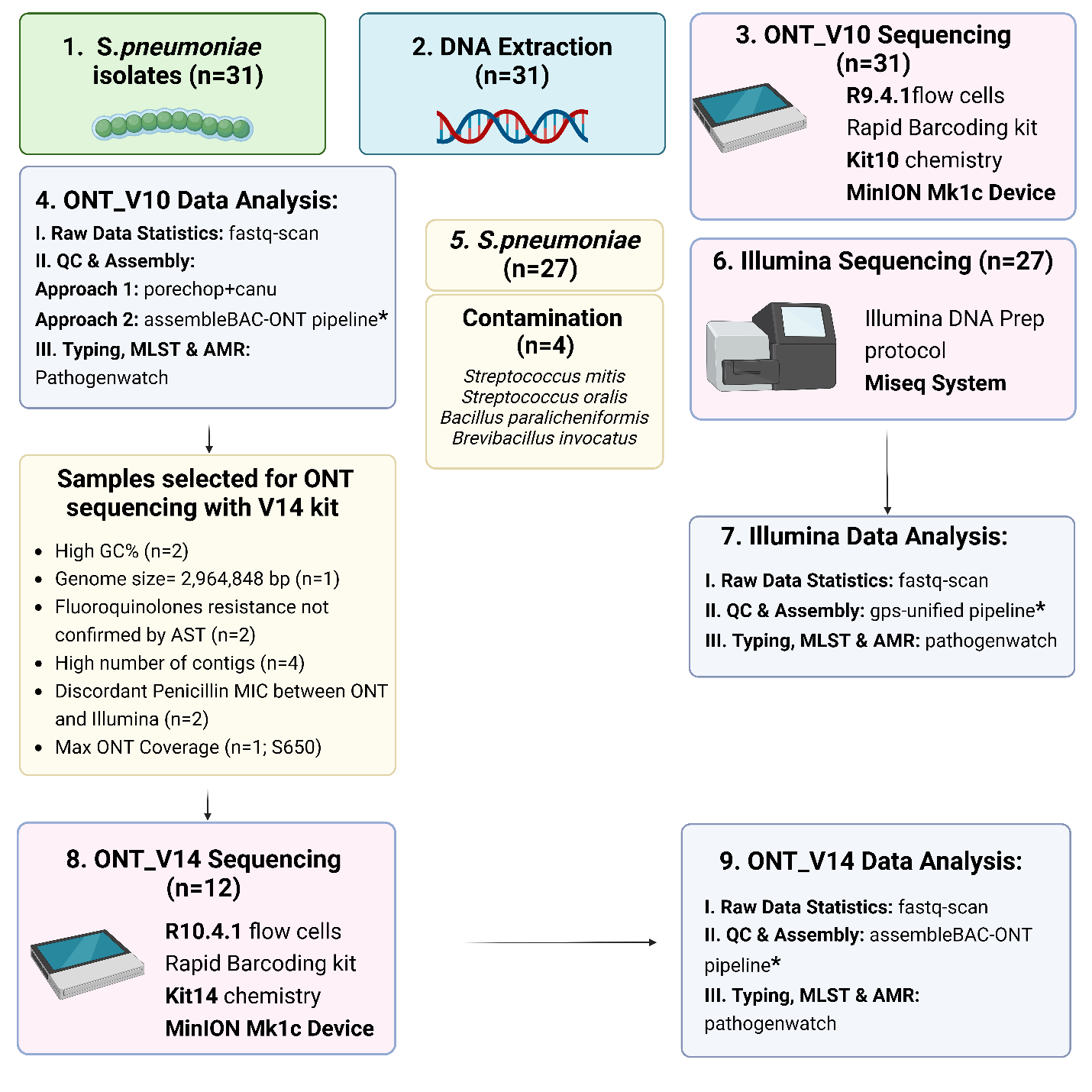
**

**Figure S1: Illustrated overview of the workflow utilized to compare sequencing outputs of Illumina, ONT_V10 and ONT_V14 techniques.**

*S. pneumoniae* bacteria were isolated from hospitalized patients with invasive pneumococcal disease (n=31) from cerebrospinal fluid (CSF) or blood (1). DNA was extracted from all samples (2) and sequenced using the ONT Mk1c system with R9.4.1 flow cells and Kit10 chemistry (ONT_V10) (3). ONT_V10 FastQ files were assembled with bioinformatic tools (porechop+canu) or with the assemble-BAC-ONT pipeline to compare the results of both approaches (4). A total of 27 isolates were identified as *S. pneumoniae* (5) and sequenced using the Illumina Miseq system (6). The gps-unified pipeline was used for the assembly of Illumina data (7). A total of 12 isolates were additionally sequenced using the ONT Mk1c system with R10.4.1 flow cells and Kit14 chemistry (ONT_V14) (8), and the resulting FastQ files were assembled with the assemble-BAC-ONT pipeline (9).

*****In addition to polished FASTA files, the assemble-BAC-ONT pipeline generates annotated files MLST data and QC reports. The gps-unified pipeline utilizes outputs assemblies, AMR, serotyping, MLST, virulence and QC data.

Abbreviations: AMR, Antimicrobial resistance; AST, Antimicrobial susceptibility testing; bp, Base pairs; MIC, Minimum inhibitory concentration; MLST, Multilocus sequence typing; ONT_V10, Oxford Nanopore Technologies (R9.4.1 flow cells/ Kit10 chemistry); ONT_V14, Oxford Nanopore Technologies (R10.4.1 flow cells/ Kit14 chemistry), QC, Quality control


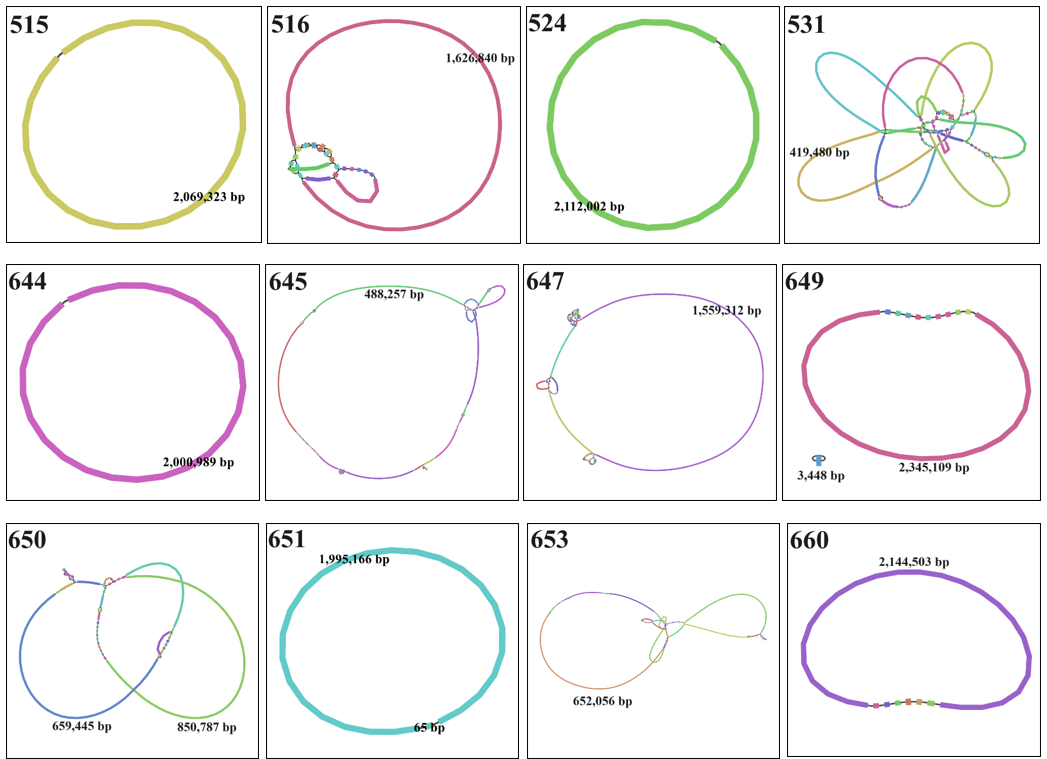


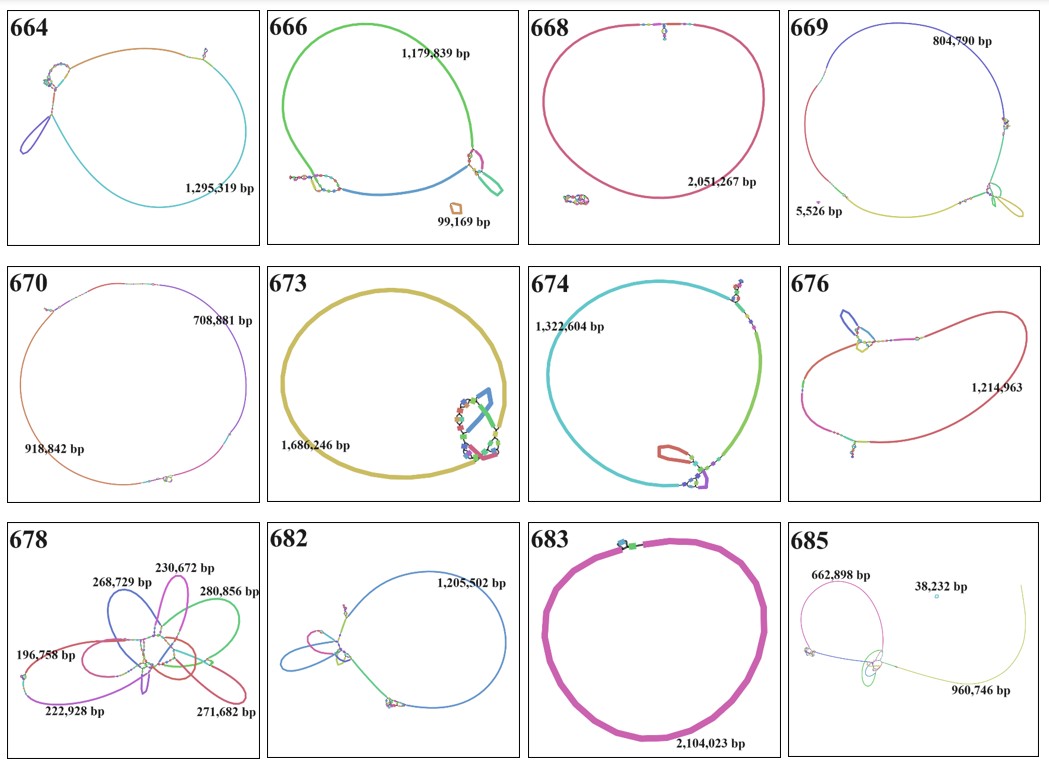

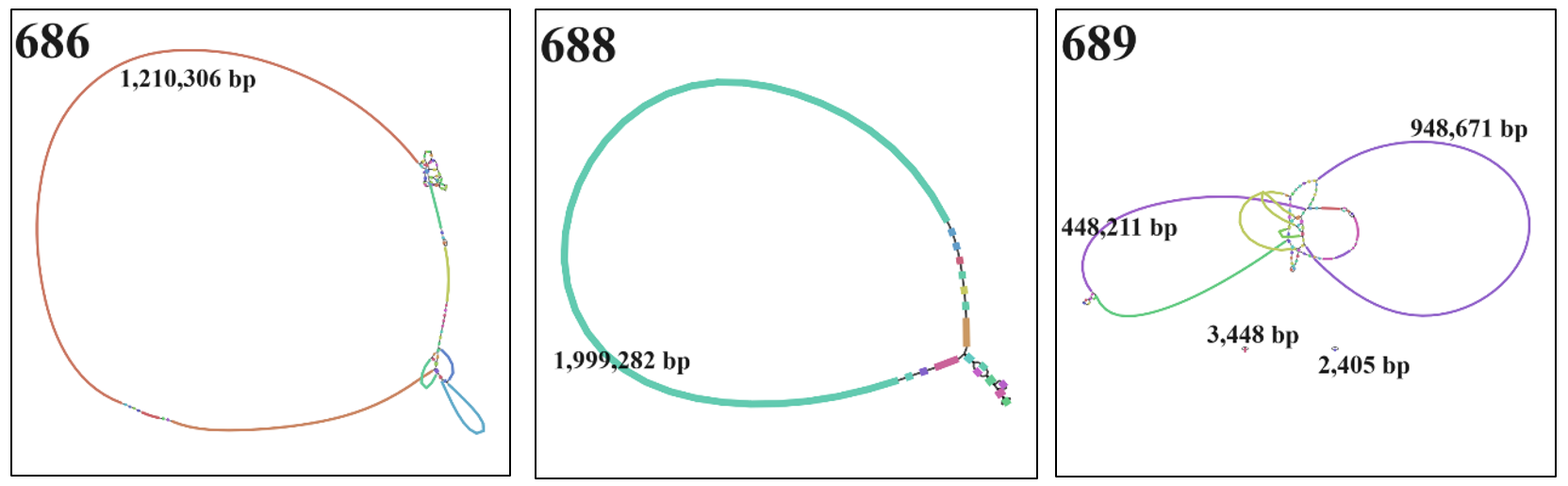


**Figure S2: Visualization of de novo assembly graphs generated by Unicycler during hybrid assembly of *S. pneumoniae* isolates (n=27) causing invasive disease.**
